# Uplift modeling to predict individual treatment effects of renal replacement therapy in sepsis-associated acute kidney injury patients

**DOI:** 10.1038/s41598-024-55653-x

**Published:** 2024-03-10

**Authors:** Guanggang Li, Bo Li, Bin Song, Dandan Liu, Yue Sun, Hongyan Ju, Xiuping Xu, Jingkun Mao, Feihu Zhou

**Affiliations:** 1 Medical School of Chinese PLA, Beijing, China; 2 Department of Critical Care Medicine, The Seventh Medical Centre, Chinese PLA General Hospital, Beijing, China; 3 Department of Automation, Tianjin University of Technology, Tianjin, China; 4 Department of Critical Care Medicine, The First Medical Centre, Chinese PLA General Hospital, Beijing, China; 5 Medical Engineering Laboratory, Chinese PLA General Hospital, Beijing, China

**Keywords:** Sepsis associated acute kidney injury, Renal replacement therapy, Individual treatment effect, Nephrology, Infectious diseases

## Abstract

Renal replacement therapy (RRT) is a crucial treatment for sepsis-associated acute kidney injury (S-AKI), but it is uncertain which S-AKI patients should receive immediate RRT. Identifying the characteristics of patients who may benefit the most from RRT is an important task. This retrospective study utilized a public database and enrolled S-AKI patients, who were divided into RRT and non-RRT groups. Uplift modeling was used to estimate the individual treatment effect (ITE) of RRT. The validity of different models was compared using a qini curve. After labeling the patients in the validation cohort, we characterized the patients who would benefit the most from RRT and created a nomogram. A total of 8289 patients were assessed, among whom 591 received RRT, and 7698 did not receive RRT. The RRT group had a higher severity of illness than the non-RRT group, with a Sequential Organ Failure Assessment (SOFA) score of 9 (IQR 6,11) vs. 5 (IQR 3,7). The 28-day mortality rate was higher in the RRT group than the non-RRT group (34.83% vs. 14.61%, p < 0.0001). Propensity score matching (PSM) was used to balance baseline characteristics, 458 RRT patients and an equal number of non-RRT patients were enrolled for further research. After PSM, 28-day mortality of RRT and non-RRT groups were 32.3% vs. 39.3%, P = 0.033. Using uplift modeling, we found that urine output, fluid input, mean blood pressure, body temperature, and lactate were the top 5 factors that had the most influence on RRT effect. The area under the uplift curve (AUUC) of the class transformation model was 0.068, the AUUC of SOFA was 0.018, and the AUUC of Kdigo-stage was 0.050. The class transformation model was more efficient in predicting individual treatment effect. A logistic regression model was developed, and a nomogram was drawn to predict whether an S-AKI patient can benefit from RRT. Six factors were taken into account (urine output, creatinine, lactate, white blood cell count, glucose, respiratory rate). Uplift modeling can better predict the ITE of RRT on S-AKI patients than conventional score systems such as Kdigo and SOFA. We also found that white blood cell count is related to the benefits of RRT, suggesting that changes in inflammation levels may be associated with the effects of RRT on S-AKI patients.

## Introduction

Sepsis is a life-threatening disease, accounting for approximately 11 million deaths per year^1^. Multiple organ dysfunction can caused by sepsis, and kidney is a target organ that most vulnerable to inflammatory damage. Observational studies have demonstrated an acute kidney injury (AKI) incidence of about 40% in patients with sepsis^2^. Conversely, sepsis contribute nearly half of the AKI occurring, and was reported as the most frequent aetiology of AKI^3,4^.

Renal replacement treatment (RRT) plays an important role in managing AKI. RRT includes methods such as intermittent hemodialysis (IHD) and continuous RRT (CRRT), with the latter typically involving continuous veno-venous hemodialysis (CVVHD) or hemodiafiltration (CVVHDF). CRRT is more popular used in intensive care settings, as it can better maintain hemodynamic stability^5^. Although RRT is widely used in AKI, there are still some questions yet to be answered^6^. Identifying the patient most likely to benefit from CRRT stands as a primary concern. Someone believes RRT may positively influence sepsis patients because RRT can modulate inflammation^7^. However, despite the removal of sepsis-related cytokines during RRT^8^, no reduction in mortality has been observed in sepsis patients, neither with high cut-off membrane filters^9^ nor with absorbing filters^10^. This highlights the importance of precise patient selection for CRRT to improve treatment outcome.

The initiation timing of CRRT is another question to be debated. A single center trial demonstrated that early RRT, compared with delayed initiation of RRT, reduced mortality over the first 90 days^11^, but other multi-center studies couldn’t find mortality decrease by early start of RRT in AKI^12^ or sepsis related AKI patients^13^. Thus, accurate evaluation of RRT benefits in S-AKI patients and initiating RRT treatment timely is essential for improving treatment outcomes.

Uplift modeling is an artificial intelligence technique that can identify the individual treatment effect (ITE) of an intervention in a population of individuals. Typically used in the field of marketing, it has been employed to identify the most vulnerable individuals who may be affected by online advertising or coupons^14^. In this study, we applied this method to estimate the ITE of S-AKI who may benefit from RRT and to identify the features of these patients.

## Material and methods

### Setting

This retrospective study utilized the Medical Information Mart for Intensive Care (MIMIC-III), a publicly available, large-scale critical care database. The database comprised 61,532 intensive care unit (ICU) admissions from 46,520 patients at the Beth Israel Deaconess Medical Center in Boston, MA, between 2001 and 2012. The data were collected from all patient admissions to various types of Intensive Care Units (ICUs) at the center during the specified period. The average length of stay in ICU was 2.1 (1.2–4.6) days, and the hospital mortality rate was 11.5%^15^. The MIMIC-III database integrates non-identifiable, comprehensive clinical data, including demographics, hourly vital signs, clinical measurements, laboratory results, treatments, and the International Classification of Diseases Ninth Revision (ICD-9) codes of diagnoses and procedures.

The data in MIMIC-III has been non-identifiable, and the institutional review boards of the Massachusetts Institute of Technology (No. 0403000206) and Beth Israel Deaconess Medical Center (2001-P-001699/14) both approved the use of the database for research. All data analysis and reporting has been performed in accordance with institutional guidelines and regulations.

### Inclusion and exclusion criteria

The inclusion criteria: (1) diagnosed as sepsis; (2) suffering from AKI. Exclusion criteria: (1) not admitted to ICU for the first time. (2) Patients age < 18. (3) End-stage renal disease (ESRD). 4. Blood potassium > 6.5 mmol/L.

To diagnose sepsis, we utilized the third sepsis definition (sepsis-3), which defines sepsis as a life-threatening condition characterized by organ dysfunction caused by a dysregulated host response to infection^16^. We screened patients with documented or suspected infection and an acute change in total Sequential Organ Failure Assessment (SOFA) score of ≥ 2. This method closely aligns with the sepsis-3 definition and has been demonstrated to be effective in the Medical Information Mart for Intensive Care III (MIMIC-III) database^17^.

AKI was defined in accordance with the Kidney Disease: Improving Global Outcomes (KDIGO) criteria^18^, which classify AKI into three stages based on urine output and serum creatinine levels. Diagnosis of AKI was confirmed if the highest KDIGO stage during the ICU stay was greater than or equal to 1. In addition to recording the baseline KDIGO stage of each patient, we also continuously documented the KDIGO stage until the initiation of RRT.

We excluded all patients with blood potassium > 6.5, which is thought to be one of the most important reasons for urgently initiating CRRT^6^. We ruled out patients not on their first admission to avoid multiple records of the same patient.

The study population was divided into two groups: the RRT group and non-RRT group, based on whether they received RRT treatment during their ICU stay.

### Primary endpoint

The primary endpoint of the study was 28-day mortality, which encompassed both in-hospital and out-of-hospital deaths.

### Statistical analysis

Continuous variables were expressed as the mean ± SD and interquartile ranges (IQR) when the data as appropriate. Student's t-test was used for normally distributed variables, while the Mann–Whitney U test was used for skewed variables. Categorical variables were presented as counts and percentages, and compared using either the chi-square test. The estimation of sample size was carried out using a power analysis based on a two-sample t-test.

To estimate the association between RRT and outcomes, as well as to select the best-matched patients for further artificial intelligence analysis, propensity score matching (PSM) was employed in our study using a greedy nearest neighbor matching algorithm. Patients were matched at a 1:1 ratio, with each RRT patient matched to a non-RRT patients, based on estimated propensity scores. The efficacy of PSM in reducing between-group differences was assessed by calculating the standardized mean difference (SMD).

### Uplift modeling

The uplift model aims to forecast the difference in outcomes between individuals who receive treatment and those who do not, while also identifying patients who are more likely to benefit from treatment. Upon reviewing previous research that employed s-learner and t-learner methods^19,20^, we found that the intrinsic logic of these studies was to indirectly model uplift. We utilized a class transformation method to create a new variable Z, where Z = 1 if a patient was in the RRT group and survived for 28 days, Z = 1 if a patient was in the non-RRT group and died within 28 days, Z = 0 if a patient was in the RRT group and died within 28 days, and Z = 0 if a patient was in the non-RRT group and survived for 28 days. We can prove that if the number of cases in the RRT group and non-RRT group was equal, then P (Z = 1│Xi) had a linear correlation with the uplift score, which can be calculated as Uplift Score = 2P (Z = 1│Xi) − 1. This method is applicable to cases where both treatment and outcome are binary variables, and single model prediction is used to achieve the conversion of prediction goals.

The validity of different models was evaluated using the adjusted qini curve, which was obtained by connecting proportion points of the adjusted qini index in different groups. The adjusted qini index was defined as following formula.

$${\text{Q}}\left(\mathrm{\varphi }\right)=\frac{{{\text{n}}}_{{\text{t}},1}\left(\mathrm{\varphi }\right)}{{{\text{N}}}_{{\text{t}}}}-\frac{{{\text{n}}}_{{\text{c}},1} (\mathrm{\varphi }){{\text{n}}}_{{\text{t}}} (\mathrm{\varphi })}{{{\text{N}}}_{{\text{t}}}{{\text{n}}}_{{\text{c}}} (\mathrm{\varphi })}$$

$\mathrm{\varphi }$ is defined as the proportion of patients ranked from highest to lowest based on their Uplift Score in either the treatment or non-treatment group. For instance, $\mathrm{\varphi }$= 0.3 represents for the top 30% uplift score patients in treatment group or none treatment group. ${{\text{n}}}_{{\text{t}},{\text{y}}=1} (\mathrm{\varphi })$ represents the number of patients in the treatment group who are predicted to survive among the given percentage of patients.${{\text{n}}}_{{\text{c}},{\text{y}}=1} (\mathrm{\varphi })$ represents the number of patients in the non-treatment group who are predicted to survive among the same percentage of patients. ${\mathrm{ N}}_{{\text{t}}}\mathrm{ and }{{\text{N}}}_{{\text{c}}}$ represents the total sample size of the treatment and non-treatment groups. The Area under the Uplift Curve (AUUC) is the area between the uplift curve and the random line, which serves as an indicator of model validity. A high AUUC corresponds to a high validity of the model.

The candidate predictors of our model included demographics, vital signs, SOFA and qSOFA scores, Kdigo stage, comorbidities and laboratory tests in the first 24 h after ICU admission. Predictor contributions were evaluated using the Shapley additive explanations (SHAP) strategy.

After modeling, we divided the patients in the validation cohort into a high benefit group and a low benefit group, and confirmed the characteristics of the high benefit group. We labeled the validation cohort according to the model, which allowed us to create a nomogram for patients who may benefit from RRT.

### Ethical approval

The clinical data used for this research was obtained from publicly available non-identifiable database, Medical Information Mart for Intensive Care (MIMIC-III), and does not require a separate ethics approval or consent obtaining process. The Institutional Review Board at the Chinese PLA General Hospital waived the review of the research plan as the data was obtained from a publicly available database with no potential violation or infringement of the ethical regulations.

## Results

### Cohort selection process

Inclusion: 1. Total Admissions: Reviewed all 61,532 ICU admissions from the MIMIC III database. 2. Sepsis Identification: Identified 25,834 admissions with sepsis using Sepsis-3 Criteria. 3. AKI Definition: Defined AKI as KDIGO ≥ 1 and 12,760 (49.39%) admissions were identified as S-AKI. Exclusion: 1. Repeat ICU Admissions: Excluded 3250 patients. 2. Underage Patients: Excluded 229 patients below 18 years. 3. ESRD Diagnosis: Excluded 403 patients based on ICD-9 codes. 4. High Blood Potassium: Excluded 589 patients with blood potassium > 6.5 mmol/L, indicative of an emergency need for RRT.

**Figure1:**
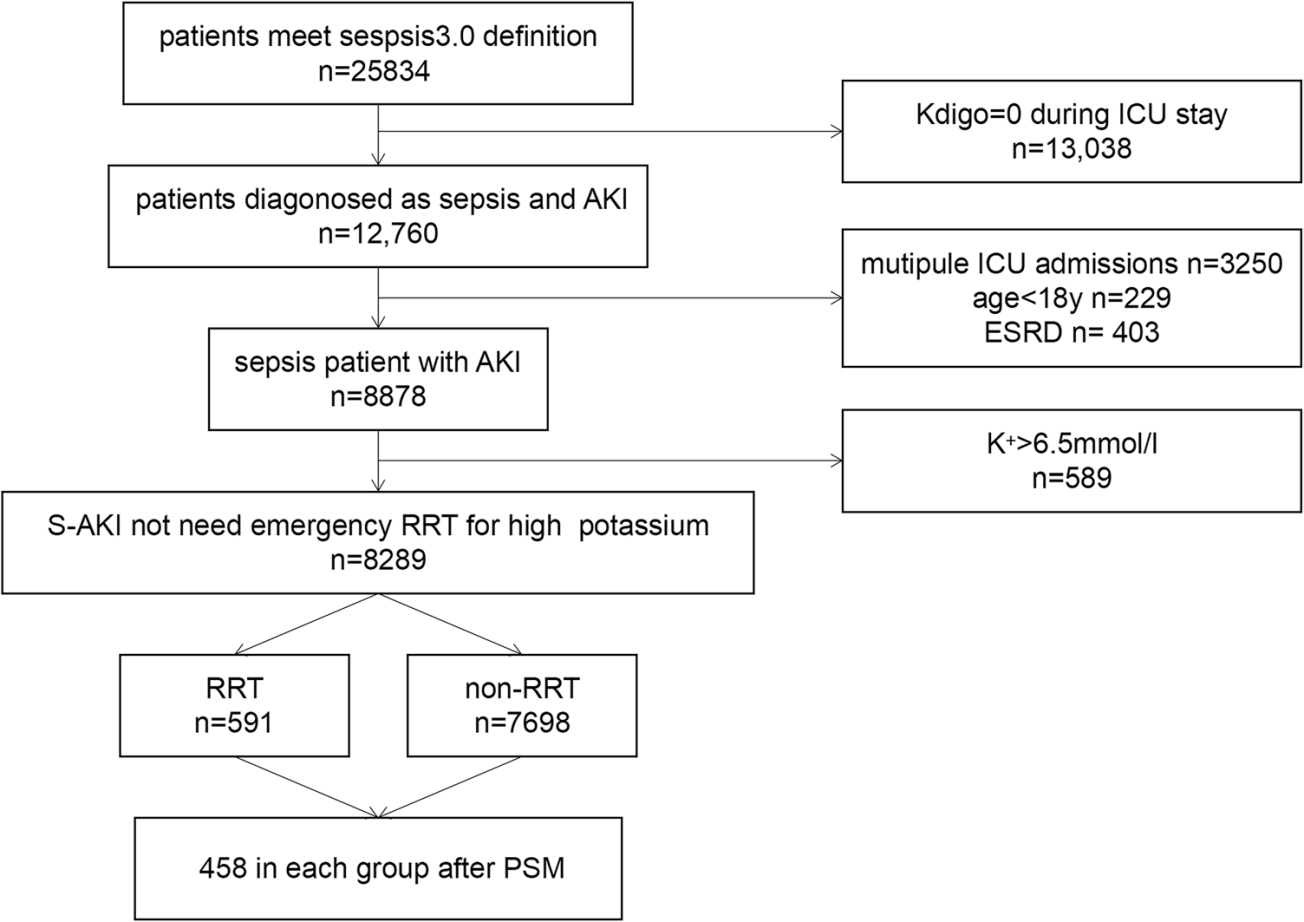
Flow chart of the study. *AKI* acute kidney injury, *RRT* renal replacement therapy, *ESRD* end-stage renal disease.

Total Eligible Admissions: 8289 ICU stays. 591 patients received RRT (RRT group) and 7698 did not received RRT (non-RRT group) (Fig. 1).

### Cohort characterization

The baseline characteristics of the two groups are shown in Table 1. Overall, the RRT group had a higher severity of illness than the non-RRT group. The SOFA score in the RRT group was 9 (IQR 6, 11) compared to 5 (IQR 3, 7) in the non-RRT group (p < 0.0001). On the first day after ICU entry, the RRT group was more likely to use mechanical ventilator (80.4% vs. 72.1%, p < 0.0001) and vasopressors (70.7% vs. 54.1%, p < 0.0001). The RRT group had higher levels of serum creatinine (364.21 ± 242.22 vs. 122.88 ± 87.52 mmol/l, p < 0.0001) and potassium (5.01 ± 0.81 vs. 4.79 ± 0.74 mmol/l, p < 0.0001) than the non-RRT group.

**Table 1:** Patients characters of RRT group, non-RRT group and non-RRT patients after PSM.

	Non-RRT (n = 7698)	RRT (n = 591)	P	RRT after PSM (n = 458)	non-RRT after PSM (n = 458)	P	SMD
Gender male (%)	4625 (60.1)	367 (62.1)	0.356	280 (61.1)	285 (62.2)	0.786	0.022
Age (mean (SD))	67.49 ± 15.62	62.61 ± 15.90	< 0.001	63.89 ± 16.20	64.51 ± 15.87	0.564	0.038
SOFA (IQR)	5 (3,7)	9 (6,11)	< 0.001	9 (6,11)	9 (6,12)	0.005	0.187
qSOFA (IQR)	2 (2,2)	2 (2,2)	0.143	2 (1,2)	2 (2,2)	0.374	0.059
Sepsis shock (%)	618 (8.0)	146 (24.7)	< 0.001	103 (22.5)	122 (26.6)	0.167	0.096
AKI-stage (%)							
0	2464 (32.0)	77 (13.0)	< 0.001	69 (15.1)	62 (13.5)	0.796	0.067
1	1509 (19.6)	88 (14.9)		71 (15.5)	66 (14.4)		
2	2893 (37.6)	139 (23.5)		125 (27.3)	136 (29.7)		
3	832 (10.8)	287 (48.6)		193 (42.1)	194 (42.4)		
SPO2 (mean ± SD)	96.90 ± 1.97	96.63 ± 2.76	0.002	96.57 ± 2.88	96.38 ± 2.99	0.350	0.062
MAP (mean ± SD)	77.58 ± 9.39	76.75 ± 10.95	0.043	76.58 ± 10.50	75.83 ± 11.16	0.301	0.068
Breath rate	19.56 ± 3.55	20.84 ± 3.94	< 0.001	20.79 ± 3.81	21.43 ± 4.21	0.016	0.159
Heart rate	86.02 ± 12.99	88.61 ± 13.12	< 0.001	88.53 ± 12.70	89.32 ± 15.10	0.393	0.057
Fluid input (IQR)	3358 (1894.5,5303.5)	2864 (1205.5,5850.25)	0.061	3344 (1385,5734.5)	3344 (2489.5,5790)	0.540	0.041
Urine output (IQR)	1554 (950,2390)	533 (181.5,1178.5)	< 0.001	763 (304.75,1500)	849.5 (351,1546.5)	0.718	0.024
Ventilator use (%)	5547 (72.1)	475 (80.4)	< 0.001	367 (80.1)	374 (81.7)	0.614	0.039
Vaspressor use (%)	4164 (54.1)	418 (70.7)	< 0.001	326 (71.2)	343 (74.9)	0.234	0.084
ICU type							
CCU (%)	946 (12.3)	100 (16.9)	< 0.001	76 (16.6)	75 (16.4)	0.181	0.166
CSRU (%)	2385 (31.0)	94 (15.9)		88 (19.2)	68 (14.8)		
MICU (%)	2484 (32.3)	285 (48.2)		205 (44.8)	237 (51.7)		
SICU (%)	1032 (13.4)	73 (12.4)		54 (11.8)	52 (11.4)		
TSICU (%)	851 (11.1)	39 (6.6)		35 (7.6)	26 (5.7)		
Co-morbidities (%)							
Chronic heart failure	2419 (31.4)	239 (40.4)	< 0.001	195 (42.6)	204 (44.5)	0.594	0.04
Chronic lung disease	1519 (19.7)	106 (17.9)	0.314	81 (17.7)	84 (18.3)	0.863	0.017
Solid tumor	247 (3.2)	9 (1.5)	0.031	9 (2.0)	10 (2.2)	1.000	0.015
Diabetes mellitus	2305 (29.9)	196 (33.2)	0.11	152 (33.2)	149 (32.5)	0.888	0.014
Hypertension	4606 (59.8)	322 (54.5)	0.012	252 (55.0)	242 (52.8)	0.551	0.044
Creatinine (mean ± SD)	122.88 ± 87.52	364.21 ± 242.22	< 0.001	3.28 ± 2.20	3.12 ± 2.16	0.273	0.072
WBC (mean ± SD)	14.57 ± 6.73	15.72 ± 8.10	< 0.001	15.66 ± 7.88	16.52 ± 8.30	0.106	0.107
Bun (mean ± SD)	28.02 ± 19.60	53.56 ± 31.25	< 0.001	48.40 ± 29.88	50.69 ± 29.88	0.247	0.077
Pt (mean ± SD)	36.91 ± 0.55	36.83 ± 0.57	< 0.001	36.83 ± 0.53	36.80 ± 0.75	0.589	0.036
Platelet (mean ± SD)	225.12 ± 102.21	205.67 ± 105.58	< 0.001	205.62 ± 102.63	205.19 ± 112.76	0.951	0.004
glucose (mean ± SD)	7.63 ± 1.76	7.79 ± 2.22	0.039	140.30 ± 37.87	140.12 ± 38.82	0.945	0.005
Sodium (mean ± SD)	140.50 ± 4.76	139.97 ± 5.40	0.009	140.06 ± 5.32	140.07 ± 6.52	0.973	0.002
Chloride (mean ± SD)	108.59 ± 6.32	105.92 ± 7.40	< 0.001	106.62 ± 7.49	106.78 ± 7.63	0.747	0.021
Lactate (mean ± SD)	3.31 ± 2.61	4.99 ± 4.43	< 0.001	4.41 ± 3.72	4.72 ± 4.16	0.239	0.078
Potassium (mean ± SD)	4.79 ± 0.74	5.01 ± 0.81	< 0.001	4.99 ± 0.80	4.93 ± 0.77	0.313	0.067
PH (mean ± SD)	7.43 ± 0.07	7.40 ± 0.09	< 0.001	7.41 ± 0.08	7.40 ± 0.09	0.368	0.06

*SOFA* sequential organ failure assessment, *qSOFA* quick SOFA, *AKI-stage* acute kidney injury stage, *MAP* mean arterial pressure, *CCU* coronary care unit, *CSRU* cardiac surgery recovery unit, *MICU* medical intensive care unit, *SICU* surgical intensive care unit, *TSICU* trauma and surgical intensive care unit, *BUN* blood urea nitrogen, *WBC* white blood cell count, *PT* prothrombin time.

To correct for imbalances between the groups, we applied 1:1 PSM. Following PSM, the sample sizes were equalized to 458 patients in each of the RRT and non-RRT groups. This adjustment resulted in a significant reduction in discrepancies post-PSM, as shown in Table 1.

Based on a sample size of 458 in each group, with mortality rates of 0.393 and 0.323 respectively, and using an alpha level of 0.05, our sample size yields a test power exceeding 99.9%.

### Primary endpoint

Prior to propensity score matching (PSM), the 28-day mortality rate was significantly higher in the RRT group compared to the non-RRT group (32.83% vs. 14.61%, p < 0.0001). After PSM, the 28-day mortality rate in the RRT group was 32.3% compared to 39.3% in the non-RRT group (p = 0.033).

### Uplift modeling

**Figure 2:**
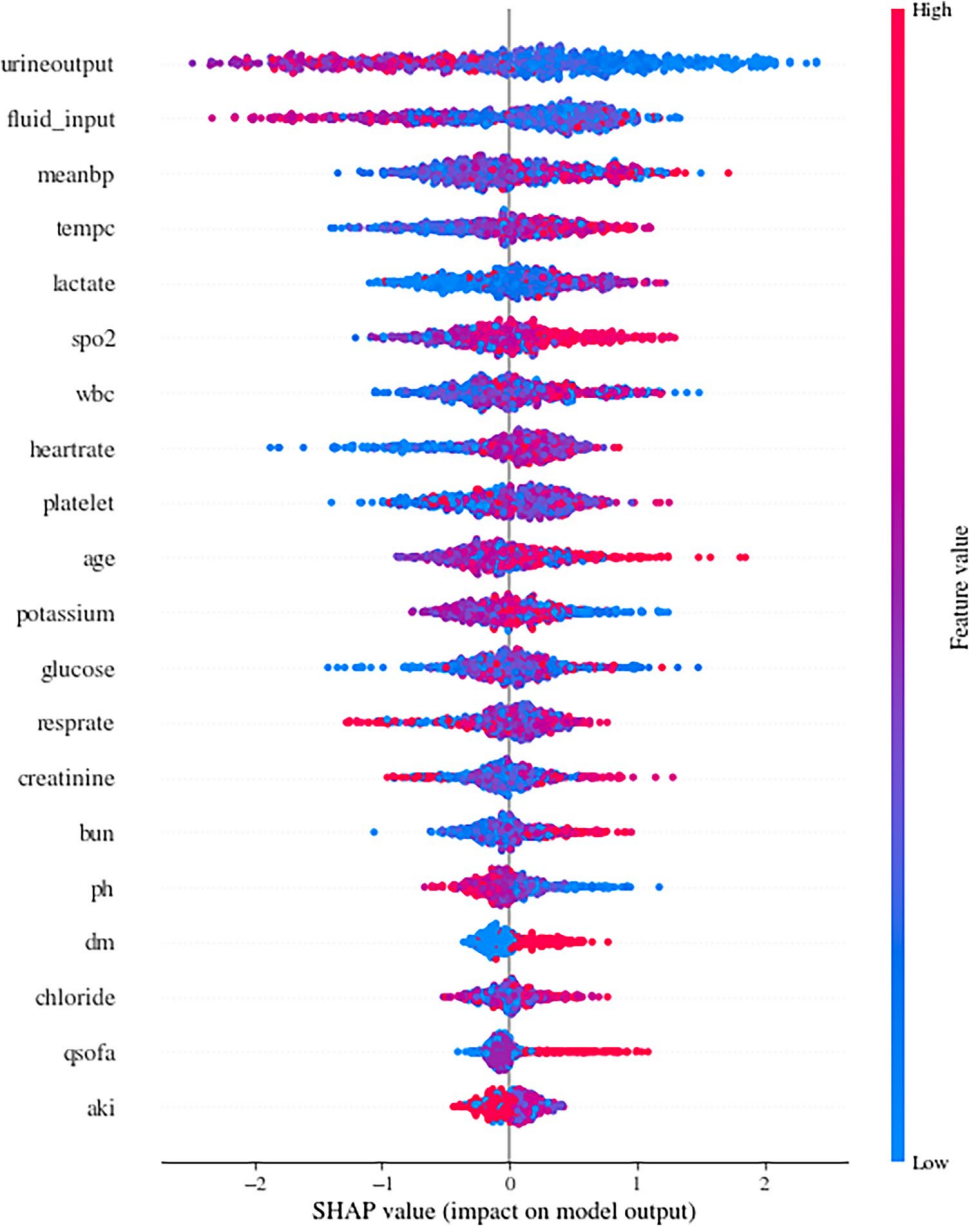
SHAP value-based predictor contribution to the ITE prediction model. Feature importance is ranked based on SHAP values. In this figure, each point represented a single observation. The horizontal location showed whether the effect of that value was associated with a positive (a SHAP value greater than 0) or negative (a SHAP value less than 0) impact on prediction. Color showed whether the original value of that variable was high (in red) or low (in blue) for that observation. For example, a low white blood cell (WBC) value had a negative impact on the outcome of patient who was receiving RRT; the “low” came from the blue color, and the “negative” impact was shown on the horizontal axis.

The enrolled patients were divided into a development cohort consisting of 640 patients and a validation cohort consisting of 276 patients. Each cohort had an equal proportion of RRT and non-RRT patients. This division, maintaining a ratio of approximately 7:3 between the training and validation cohorts, is a common practice in such studies. After performing uplift modeling in the development cohort, several parameters were included in the model. Figure 2 shows that urine output, fluid input, mean blood pressure, body temperature, and lactate were the top five factors that most influenced the RRT effect. In addition, other predictors such as age,, SpO_2_, white blood cell count, ph, platelet count and potassium also had an impact on the RRT effect.

### Modeling validity

**Figure 3:**
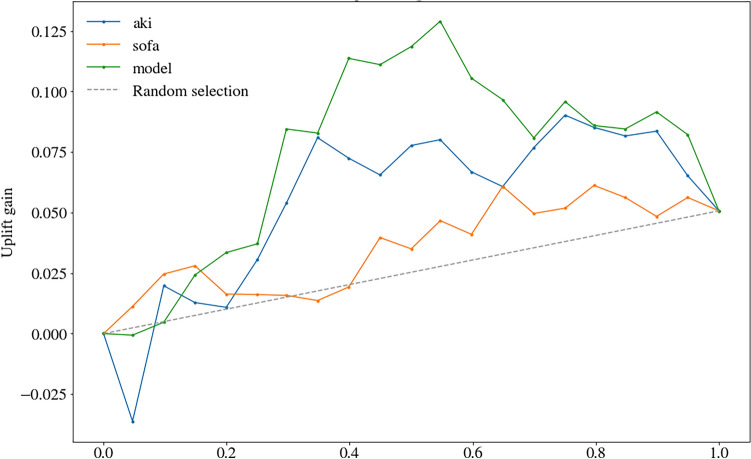
Qini curve. The area under the uplift curve (AUUC) represents the area between a specific model curve and the random line, and serves as an indicator of model validity. A higher AUUC indicates higher validity, and an AUUC > 0 indicates model validity greater than random selection. In this study, the AUUC for the class transformation model was 0.074, for SOFA it was 0.022, and for Kdigo-stage it was 0.031.

The modified qini curve was used to compare the predicting ability of our model with conventional scoring systems including SOFA and Kdigo-stage. As shown in Fig. 3, the area under the uplift curve (AUUC) of the class transformation model was 0.068, the AUUC of SOFA was 0.018, and the AUUC of Kdigo-stage was 0.050. The class transformation model was more efficient in predicting the individual treatment effect of RRT than conventional scores.

### Characters difference between high benefit and low benefit patients

**Figure 4:**
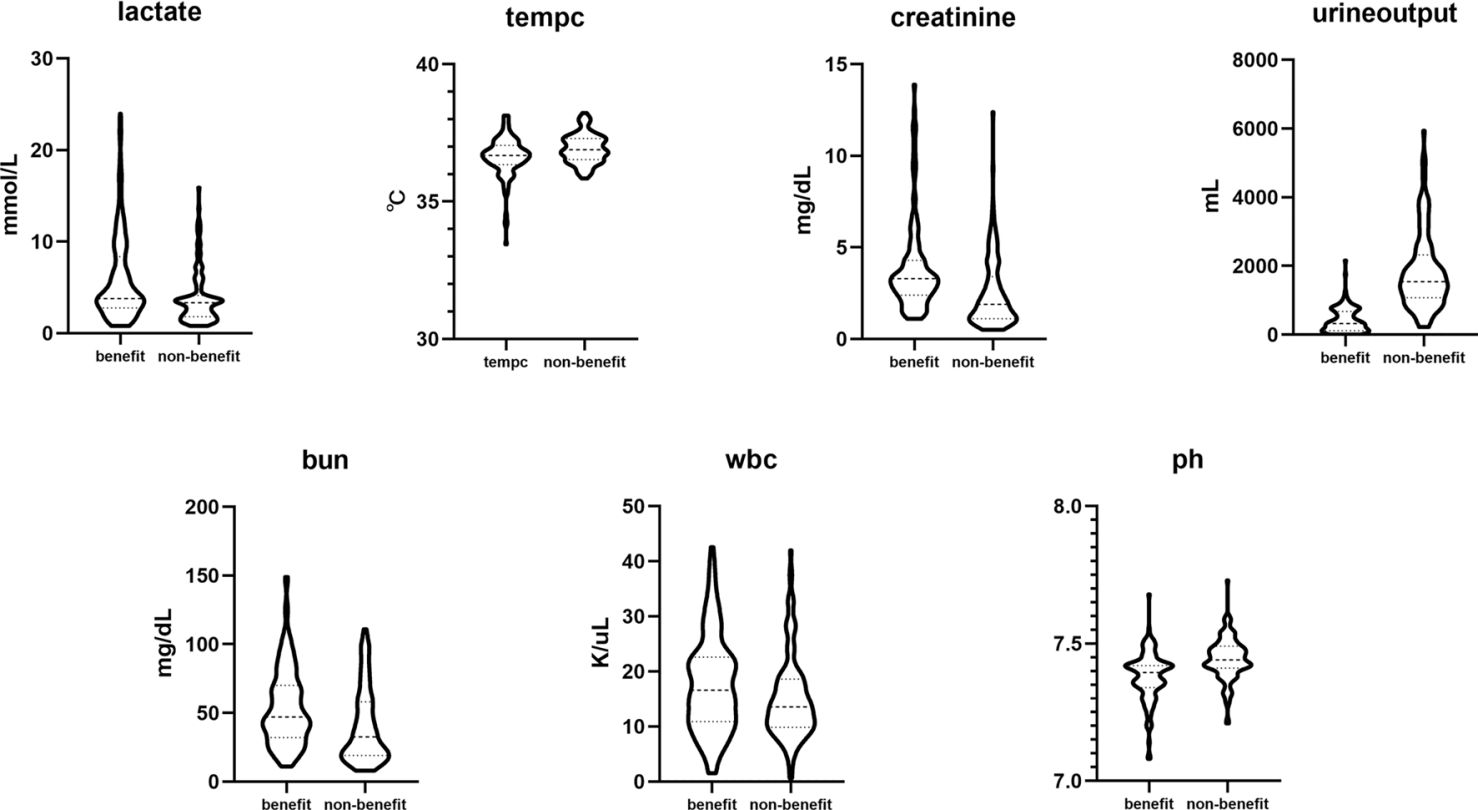
Different characteristics between RRT benefit and non-benefit patients. The seven parameter had statistic difference (p < 0.05) between two groups were lactate, body temperature, creatinine, urine output of first 24h admission in ICU, BUN, white blood count (WBC) and ph.

We classified the patients in the validation group into high benefit group (n = 138) and low benefit group (n = 138), using class transformation. The high benefit individuals possessed different characteristics from low benefit individuals. As shown in Fig. 4, the high benefit group and low benefit group differed in renal-related indicators, including urine output, creatinine, BUN, pH, and chloride. Additionally, white blood cell count was found to be related to the effect of RRT.

**Figure 5:**
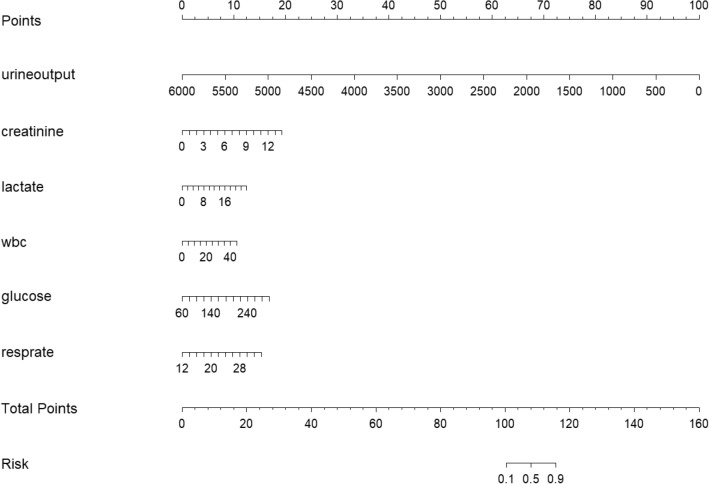
Nomogram for RRT benefit probability. Each parameter has a range of values that can be used to find different corresponding numerical values on the line segment. The sum of all the parameter values yields a score which can be used to calculate the probability of benefit from RRT therapy. *wbc* white blood cell.

We constructed a logistic regression model with the benefit status of patients (benefit vs. non-benefit) as the dependent variable. Six factors with statistical significance between groups were selected as the independent variables, including urine output, creatinine, lactate, white blood cell count, glucose, respiratory rate (Table 2). We then built a nomogram of the risk predictive value of the model. As showed in Fig. 5. The nomogram illustrates the contribution rate of each risk index by the length of the corresponding line segment, providing a visual and practical representation of our model.

**Table 2:** Logistic regression analysis of S-AKI patients benefit from RRT.

Variable	OR	95% CI	p value
Urine output	0.99	0.99–0.99	< 0.001
Creatinine	1.49	1.16–1.92	0.002
Lactate	1.16	1.01–1.33	0.034
White blood cell	1.07	1.01–1.13	0.019
Glucose	1.02	1.01–1.04	0.014
Respiratory rate	1.22	1.07–1.40	< 0.004

*SOFA* sequential organ failure assessment, *WBC* white blood cell count.

## Discussion

The optimal approach for administering RRT to AKI patients remains a critical issue in critical care medicine^6^. RRT has been suggested to down regulate immune responses in sepsis, introducing a promising dimension in the therapeutic approach for S-AKI patients. In this study, we classified S-AKI patients into RRT and non-RRT groups and compared their baseline characteristics and 28-day mortality rates, which were initially unbalanced. As expected, the RRT group had greater severity and had higher 28-day mortality rates. This reflecting the clinical reality that clinicians often tend to administer RRT to more severe patients. After PSM, the imbalance between the two groups was significantly reduced. These findings suggest the need for a more precise characterization of patients who can benefit from RRT. To address this issue, we utilized the uplift technique to model the treatment effect of RRT and identify S-AKI patients who may derive the most benefit from RRT. Our model outperformed traditional clinical indicators, such as the Kdigo grade, which are typically used to determine the need for RRT in S-AKI patients. Our model identified several parameters that were associated with the benefit from RRT. Notably, our findings suggest that certain inflammation-related factors, such as White Blood Cell (WBC) count is associated with improved outcomes in patients receiving RRT. Moreover, we developed a nomogram to assist in determining whether to initiate RRT in S-AKI patients.

In recent years, several randomized controlled studies (RCT) have been conducted to investigate the optimal timing of RRT in patients with AKI^12,13,22^, with one study specifically focusing on patients with septic AKI^13^. The findings from most of these studies indicate no significant difference in mortality between patients who received early RRT and those who received late RRT. For instance, the IDEAL-ICU trial recruited septic shock patients with AKI and showed no significant difference in overall mortality at 90 days between patients assigned to early or delayed RRT strategies^13^. Although RCTs are the gold standard for assessing treatment effects, they have limitations, such as stringent inclusion criteria and high cost^23^. For instance, the IDEAL-ICU trial, which was conducted in 29 ICUs over four years, screened 3753 patients, of whom 1728 met the inclusion criteria, but only 488 were randomized, with 246 patients in the early RRT group and 242 patients in the delayed RRT group^13^. The difficulties RCTs face in enrolling critically ill patients make the use of Real-World Evidence (RWE) data for analysis even more meaningful. RWE data, as a vital complement to RCTs, has been acknowledged in medical research^24^. In our study, we focused on S-AKI, which is a life-threatening subtype of AKI associated with sepsis. Our study employed real-world big data and enrolled 458 patients in the RRT group, and an equal number of 458 patients in the non-RRT group. Our study aims to supplement the gaps in RCTs and suggest possible approaches for administering RRT to S-AKI patients.

Prospective research has also investigated the indications for RRT in AKI^25–28^. However, we identified major selection bias in these studies. All these studies analyzed only patients who received RRT, patients who recovered from severe acute kidney injury without ever receiving RRT were excluded. As these patients might be more likely to have a good prognosis compared with those who undergo RRT, omission of these patients constitutes a major selection bias. Some clinicians have also raised this point^29^. This selection bias is also present in research that uses machine learning algorithms^30^. The models built by such studies can only predict the patients who have received RRT but not all patients who may need RRT. In our study, we included all patients meeting the criteria of Sepsis-3 and KDIGO stage > 1, regardless of whether they received RRT or not. The model we built has a wider scope of use than other prospective studies and can predict the expected treatment effect of all S-AKI patients.

The majority of studies on the timing of RRT have used renal function as the primary indication for early treatment, based on Kdigo^12,22^ or RIFLE staging^13^. However, relying solely on renal function as an indication may not provide a comprehensive understanding of the intrinsic nature of S-AKI. It is widely acknowledged among clinicians that modifying the immune response through RRT may have a positive impact on sepsis, as demonstrated by several studies^31,32^. We excluded all patients with blood potassium levels greater than 6.5, aiming to extensively eliminate those who urgently require RRT treatment. We believe this exclusion criterion enhances the practical significance and ethical alignment of our model. However, we did not exclude patients with volume overload, as this is a common state in sepsis treatment due to the essential role of fluid resuscitation.

Our research suggests that in addition to renal function, other indicators such as inflammation markers should be considered when making a decision about RRT for S-AKI. Figure 4 demonstrates that, in addition to renal-related indicators, several other factors are associated with the response to RRT. These include disease severity, as indicated by SOFA scores; volume overload, reflected in SpO2 levels; and inflammation level, evidenced by white blood cell count. This result indicates that when making a decision about starting RRT, factors such as the severity of sepsis and inflammation level should also be taken into account.

Applying the results of an RCT to individual patients should be done with caution, as there may be heterogeneity between individual patients that was not captured in the RCT^33,34^. Critical ill patients have a high degree of heterogeneity in disease states, which can generate heterogeneity in their response to treatment^35^. As shown in Table 1, patients who received RRT were much more severe, reflecting the real-world situation in which doctors are more likely to initiate RRT in severe cases. After balancing the differences between the RRT and non-RRT groups using PSM, we utilized the data for ITE analysis to identify characteristics related to treatment effects for each individual in high heterogeneity diseases like AKI. Uplift modeling has been used in AKI patients to identify those who can benefit from electronic alerts^36^. Our uplift modeling approach also produced a nomogram of the factors influencing the RRT effects on S-AKI patients. This work may be useful to clinicians when making decisions about whether to perform RRT on a S-AKI individual.

Randomized data can be applied to Uplift Modeling, such as data from RCTs, which can accurately distinguish the potential effects of treatments on different individuals^37^. However, randomized data is not always available. Non-randomized data, after being processed, can also be applied to uplift modeling^38^. For example, undersampling is an approach used in this context. It works by reducing the number of observations in the majority class, thus addressing the imbalance between different treatment groups. PSM is a method of under sampling and a common approach for applying non-randomized data in uplift modeling. Our study employed PSM to balance the baseline characteristics between the RRT and non-RRT groups, allowing this real-world data set to be applied to uplift modeling. This approach addresses the issue of insufficient randomized data for RRT treatment. Nonetheless, it is essential to note that applying non-randomized data to uplift modeling requires careful consideration of the potential impact that imbalances between groups may have on the results.

Our research has some limitations. Firstly, the training and test sets were all taken from the MIMIC database, which may limit the generalizability of our results. We looked into other public databases, such as the eICU Collaborative Research Database^39^ but found that they did not have sufficient data to enroll the model. Secondly, the indicators included in our model were conventional parameters, and some novel biomarkers associated with AKI, such as NGAL and KIM-1^40^, were not studied. More research is needed to investigate the relationship between biomarkers and RRT treatment effects in the future.

## Conclusion

Uplift modeling can better predict the ITE of RRT on S-AKI patients than conventional score systems such as Kdigo and SOFA. These findings are highly relevant to clinical practice, as they offer valuable insights into the identification of subgroups of S-AKI patients who are most likely to benefit from RRT.

## Data Availability

The raw data for this study was obtained from the MIMIC-III database, which can be accessed through the following website link: https://mimic.physionet.org.

## Abbreviations

AKI: Acute kidney injury
AUUC: Area under the uplift curve
CRRT: Continuous renal replacement therapy
CVVHD: Continuous veno-venous hemodialysis
CVVHDF: Continuous veno-venous hemodiafiltration
ESRD: End-stage renal disease
ICD-9: International classification of diseases ninth revision
ICU: Intensive care unit
IHD: Intermittent hemodialysis
IQR: Interquartile range
ITE: Individual treatment effect
KDIGO: Kidney disease: improving global outcomes
MIMIC-III: Medical information mart for intensive care III
PSM: Propensity score matching
RCT: Randomized controlled studies
RRT: Renal replacement therapy
SD: Standard deviation
S-AKI: Sepsis-associated acute kidney injury
SMD: Standardized mean difference
SOFA: Sequential organ failure assessment
